# Acid mediates a prolonged antinociception via substance P signaling in acid-induced chronic widespread pain

**DOI:** 10.1186/1744-8069-10-30

**Published:** 2014-05-21

**Authors:** Wei-Nan Chen, Chih-Cheng Chen

**Affiliations:** 1 Graduate Institute of Life Sciences, National Defense Medical Center, Taipei 114, Taiwan; 2 Institute of Biomedical Sciences, Academia Sinica, Taipei 115, Taiwan; 3 Taiwan Mouse Clinic-National Comprehensive Mouse Phenotyping and Drug Testing Center, Academia Sinica, Taipei 115, Taiwan; 4 128 Academia Road, Section 2, Taipei 115, Taiwan

**Keywords:** Antinociception, ASIC3, TRPV1, Muscle pain, Nociceptor

## Abstract

**Background:**

Substance P is an important neuropeptide released from nociceptors to mediate pain signals. We recently revealed antinociceptive signaling by substance P in acid-sensing ion channel 3 (ASIC3)-expressing muscle nociceptors in a mouse model of acid-induced chronic widespread pain. However, methods to specifically trigger the substance P antinociception were still lacking.

**Results:**

Here we show that acid could induce antinociceptive signaling via substance P release in muscle. We prevented the intramuscular acid-induced hyperalgesia by pharmacological inhibition of ASIC3 and transient receptor potential V1 (TRPV1). The antinociceptive effect of non-ASIC3, non-TRPV1 acid signaling lasted for 2 days. The non-ASIC3, non-TRPV1 acid antinociception was largely abolished in mice lacking substance P. Moreover, pretreatment with substance P in muscle mimicked the acid antinociceptive effect and prevented the hyperalgesia induced by next-day acid injection.

**Conclusions:**

Acid could mediate a prolonged antinociceptive signaling via the release of substance P from muscle afferent neurons in a non-ASIC3, non-TRPV1 manner.

## Findings

### Background

Substance P (SP) is a neuropeptide released from nociceptors to mediate pain transmission centrally and neurogenic inflammation peripherally [1,2]. Our recent study showed that release of SP from muscle nociceptive nerve fibers was antinociceptive in a mouse model of acid-induced chronic widespread pain [3]. The unexpected antinociceptive role of SP was found exclusively in acid-sensing ion channel 3 (ASIC3)-expressing muscle nociceptors: SP acts on neurokinin 1 (NK1) receptors to reduce acid-induced depolarization via activation of an M-type potassium channel that is G protein-independent but tyrosine kinase-dependent. The inhibitory effect of SP could prevent the development of chronic widespread pain induced by repeated intramuscular acid insults.

Acid is effective in causing chronic muscle pain via activation of ASIC3 or transient receptor potential V1 (TRPV1) in muscle nociceptors [4-8]. The mouse model of acid-induced chronic widespread muscle pain was developed to mimic the clinical symptoms of fibromyalgia, exhibiting both chronic widespread pain and autonomic dysfunction [4,9]. In this model, 2 injections (separated by 2 to 5 days) of pH4.0 acid saline to one side of the gastrocnemius muscle caused bilateral, long-lasting referred hyperalgesia in mouse hind paws. The first acid injection produces only transient hyperalgesia that diminished in 24 h, and the second acid injection 2 to 5 days later to the same muscle causes long-lasting hyperalgesia that lasts for more than 4 weeks. In mice lacking SP signaling by genetic ablation or pharmacological blockade in muscle, a single intramuscular acid injection is enough to produce long-lasting hyperalgesia [3]. In contrast, an additional boost of SP with the second acid injection prevents the development of chronic widespread pain in wild-type mice that received the first acid injection.

The physiological mechanism underlying the SP-mediated inhibition of ASIC3 signaling might be therapeutically useful, because application of an NK1 agonist prevented the development of long-lasting hyperalgesia induced by a second acid injection; however, the beneficial effect of endogenous SP release was lost during the second acid challenge [3].

Here, we aimed to probe possible ways to trigger and enhance SP analgesia in the mouse model of chronic widespread pain.

## Results

**Figure 1:**
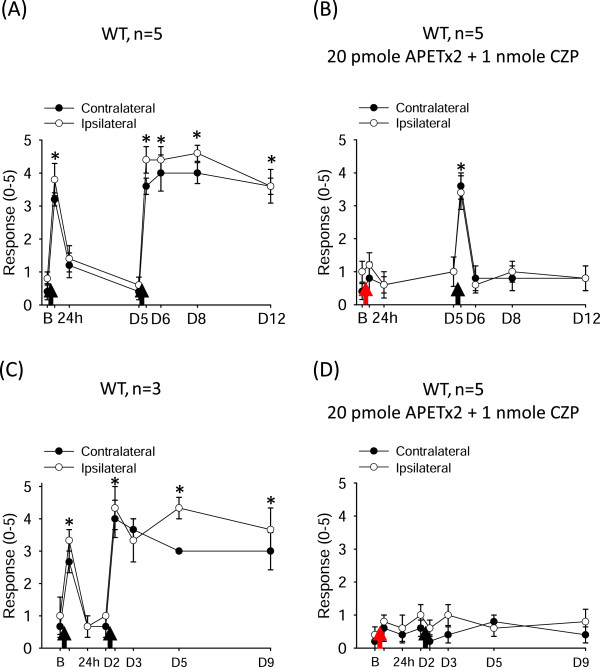
**Acid-induced prolonged antinociceptive signaling in muscle.** The withdrawal responses of mouse hind paws to a 0.2-mN bending force in mice before and after intramuscular acid injection in the gastrocnemius muscle. **(A)** In the dual acid injection scheme, mice showed transient hyperalgesia after the first intramuscular acid injection and chronic hyperalgesia after a second acid injection spaced 5 days apart. **(B)** A mix of acid saline with APETx2 and capsazepine abolished the transient hyperalgesia with the first injection and prevented the development of chronic hyperalgesia induced by the second acid injection 5 days later. **(C,D)** With the first acid injection, acid induced a prolonged antinociceptive effect lasting for 2 days, with ASIC3 and TRPV1 blocked by APETx2 and capsazepine, respectively, and a second acid injection at day 2 could not induce any hyperalgesic effect. Black arrows indicate when mice received the intramuscular acid injection. Red arrows indicate when mice received the co-injection of acid with APETx2 and capsazepine. B, baseline on day 0; D, day; WT, wild-type mice; CZP, capsazepine. *P < 0.05 compared with the response at baseline.

Although ASIC3 and TRPV1 play major roles in acid-induced nociception in muscle pain models, accumulating evidence has shown that a subset of muscle nociceptors express other acid sensors, such as ASIC1a, ASIC1b, and ASIC2a [10-13]. Thus, we examined whether acid had other biological effects in muscle in a non-ASIC3, non-TRPV1 signaling pathway. We tested this hypothesis in the Sluka acid-induced chronic widespread pain model. We mixed acid saline (pH 4.0) with APETx2 (a selective ASIC3 antagonist) [14] and capsazepine (a selective TRPV1 antagonist) [15] for the first intramuscular injection of the dual acid-injection scheme. As expected, the mixture of APETx2 and capsazepine abolished the acid-induced transient hyperalgesia with the first injection; a second acid injection 5 days later induced only transient hyperalgesia (Figure 1A,B). The second acid injection even failed to induce transient hyperalgesia when administered 1 or 2 days after the first injection (Figure 1C,D). A possible explanation is that the acid had a prolonged antinociceptive effect in muscle via non-ASIC3, non-TRPV1 signaling in muscle afferent neurons.

**Figure 2:**
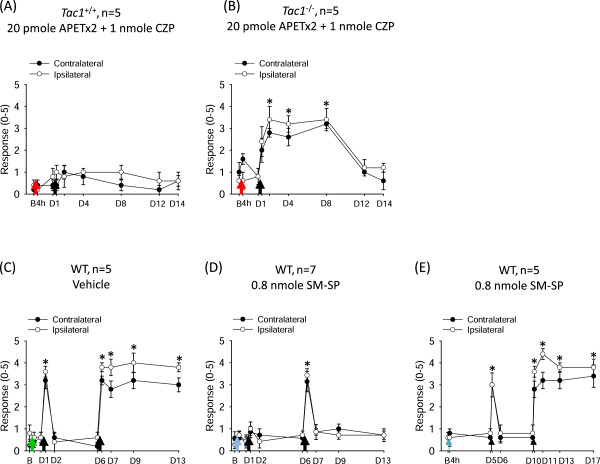
**Substance P mediates the acid-induced prolonged antinociception in acid-induced chronic widespread pain model. (A,B)** APETx2 and capsazepine prevented acid-induced chronic hyperalgesia in tachykinin 1-positive (*Tac1*^*+/+*^) mice **(A)** but not *Tac1*^*−/−*^ mice **(B)** when the second acid injection was given the next day. **(C)** Pretreatment with pH 7.4 saline did not affect the acid-induced hyperalgesia in wild-type mice. **(D)** Pretreatment with SM-SP abolished the acid-induced transient hyperalgesia the next day and prevented the development of chronic hyperalgesia induced by a second acid injection 5 days later. **(E)** Pretreatment with SM-SP 5 days before the acid injection did not affect the acid-induced transient and chronic hyperalgesia in the dual acid injection scheme. Black arrows indicate when mice received the intramuscular acid injection. Red arrows indicate when mice received the co-injection of acid with APETx2 and capsazepine. Green arrow indicates when mice received a pretreatment of pH 7.4 saline 1 day before the acid injection. Blue arrows indicate when mice received a pretreatment of SM-SP 1 or 5 days before acid injection. B, baseline on day 0; D, day; WT, wild-type mice; CZP, capsazepine; SM-SP, [Sar^9^,Met(O_2_)^11^]-substance P; Tac1, tachykinin 1. *P < 0.05 compared with the response at baseline.

We next probed whether the prolonged antinociceptive effect of non-ASIC3, non-TRPV1 acid signaling was mediated by SP release. We examined the effect of APETx2 and capsazepine on acid-induced hyperalgesia in mice lacking the tachykinin 1 gene (*Tac1*^*−/−*^; no SP and no neurokinin A production). APETx2 combined with capsazepine could abolish the acid-induced transient hyperalgesia during the first injection in *Tac1*^*−/−*^ mice as in their wild-type littermates (Figure 2A,B). Intriguingly, *Tac1*^*−/−*^ mice showed acid-induced chronic hyperalgesia (for 1 week) when the second acid injection was given the next day. Therefore, SP may play a key role in acid-induced prolonged antinociception in non-ASIC3, non-TRPV1 signaling in muscle afferent neurons. If so, SP alone should be able to reproduce the prolonged antinociceptive effect in the acid-induced chronic widespread pain model. Indeed, on pretreatment with an NK1-selective agonist, [Sar^9^,Met(O_2_)^11^]-substance P (SM-SP), 1 day before the acid injection, acid no longer induced hyperalgesia (Figure 2C,D). Just like non-ASIC3, non-TRPV1 acid signaling, the SM-SP-induced prolonged antinociceptive signaling did not last for 5 days. Five days after pretreatment with SM-SP, dual acid injections 5 days apart induced transient hyperalgesia at 4 h after the first acid injection and long-lasting hyperalgesia after the second acid injection at 5 days (Figure 2E).

## Conclusions/Discussion

Although acid is an effective algogen causing pain, especially muscle pain [16,17], we report here an antinocieptive role for acid in a mouse model of chronic widespread pain. This acid-mediated antinociceptive pathway might act via a subset of acid-sensitive muscle afferent neurons that are neither ASIC3- nor TRPV1-positive to release SP. This finding expands our recent knowledge of SP antinociceptive signaling, which could have a prolonged inhibitory effect, lasting for 2 days, on muscle nociceptors.

Previously, we found that SP activated M-type potassium channels in muscle nociceptors in a G protein-independent manner and thus inhibited the ASIC3-induced inward current. The inhibitory effect of SP was reproduced *in vivo*, with abolished acid-induced chronic widespread pain on co-injection of SP with acid saline into the gastrocnemius muscle [3]. In the acid-induced chronic widespread pain model, acid signaling seems to dominantly activate ASIC3 or TRPV1 to evoke a referred hyperalgesia in mouse hind paws and nociceptor priming [4,8]. The results of the current study suggest that acid also activates receptors or ion channels in muscle afferent neurons that express neither ASIC3 nor TRPV1 to elicit the release of SP and limit the hyperalgesia to a transient phase. The possible acid sensors might be other members of the ASIC family (e.g., ASIC1a and ASIC2), which are also expressed in muscle afferent neurons [12]. Alternatively, acid might activate a group of proton-sensing G protein-coupled receptors (e.g., G2A, GPR4, OGR1, and TDAG8) that are functionally coupled with ASIC3 [18-20]. Future studies should identify the molecular identity of the antinociceptive acid sensor(s), which will shed new insight for the development of analgesic drugs targeting chronic widespread muscle pain, such as in fibromyalgia.

We found that muscular SP signaling, endogenously released from muscle afferent neurons by acid challenge or exogenously injected, could silence muscle nociceptors from further firing in 2 days. How the G-protein-independent signaling with SP antinociception could last for 2 days remains unknown. The G protein-independent SP/NK1 signaling in muscle nociceptors might be as complicated as its G protein-dependent pathway in other cell types [21,22]. Nevertheless, this knowledge is clinically useful because we can then develop strategies to prevent the development of chronic muscle pain caused by repeated muscle injury or ischemic insult.

## Material and methods

### Animals

We used male C57/BL6 mice (8 to 12 weeks old). All procedures were approved by the Institutional Animal Care and Use Committee of Academia Sinica and followed the *Guide for the Use of Laboratory Animals* (National Academy Press, Washington, DC). Mice lacking tachykinin 1, the gene encoding SP (*Tac1*^*−/−*^), were generated as previously described [23]. *Tac1*^*+/+*^ and *Tac1*^*−/−*^ mice were offspring of congenic C57BL6 *Tac1*^*+/−*^ intercrosses.

### Pain behaviors

The mouse model of acid-induced chronic widespread pain induced by repeated intramuscular acid injection was as described [4], with modification, and described previously [3]. Briefly, mice received an intramuscular injection in the gastrocnemius muscle of acid containing 20 μL acid saline (pH 4.0) with or without APETx2 (20 pmol; Alomone, Jerusalem, Israel) and capsazepine (1 nmol; Torcis, Avonmouth, UK). Mechanical hyperalgesia of hind paws was measured by application of a 0.2-mN von Frey filament. The experimenter conducting the von Frey test had no information about the mouse genotypes or drug injections. In some studies, mice received pretreatment with an intramuscular injection of pH 7.4 saline or 40 μM [Sar^9^,Met(O_2_)^11^]-substance P (SM-SP; Sigma, St. Louis, MO) at 1 or 5 days before the acid injection.

### Data analysis

The Mann–Whitney U test was used to compare withdrawal responses to the von Frey filament application in mice before acid or pH 7.4 saline injection (baseline) and at each time after intramuscular injection of acid (with or without drugs). P < 0.05 was considered statistically significant.

## Abbreviations

ASIC3: Acid-sensing ion channel 3; SP: Substance P; TRPV1: Transient receptor potential V1.

## Competing interests

The authors declare that they have no competing interests.

## Authors’ contributions

All authors have read and approved the final manuscript. WNC conducted the experiments and analyzed the data. CCC designed the study and wrote the manuscript.
